# Leading the Pack: Next‐Generation Batteries for Humanoid Robotics

**DOI:** 10.1002/advs.76736

**Published:** 2026-07-29

**Authors:** Matthew Bergschneider, Jiaqi Ke, Jin Luo, Laisuo Su, Thomas Campbell, Yue Zhou, Kyeongjae Cho

**Affiliations:** ^1^ Department of Materials Science and Engineering University of Texas at Dallas Richardson Texas USA; ^2^ Department of Mechanical Engineering University of Texas at Dallas Richardson Texas USA; ^3^ LEAP Manufacturing Evergreen Colorado USA

**Keywords:** batteries, humanoid robotics, power sources

## Abstract

Transitioning from demonstration to commercial deployment of humanoid robots offers the potential to accelerate multiple industries. As mechatronic and software demands increase, uninterrupted application requires a quadrupling of battery system volumetric and gravimetric capacity. First considered here are the unique challenges of the humanoid robot platform. The upper bounds of further optimized current generation battery materials, electrodes (electrode coatings, electrolytes, etc.), and battery design (cylindrical, pouch, or prismatic) can narrow the gap of practical capacities toward theoretical limits, but only so far. Application cases indicate energy demands of 10+ kWh, which will necessitate next‐generation batteries with specific energy > 1000 Wh L^−1^ (Li metal or Li alloy anodes, solid electrolytes, and/or conversion chemistries: metal‐air or Li‐S) and corresponding re‐evaluation of cell and pack design for integration into humanoid robot systems. Stopgap improvements and standardizations in battery pack technologies are essential for swappable batteries to bypass interim capacity limitations. This note provides an overview of the challenges and opportunities in energy storage of humanoid robots, with the goal of catalyzing researchers and industry to advance this critical technology.

## Graphical abstract

Humanoid robots challenge traditional and anticipated energy technologies. Humanoid robot benchmarks of runtimes and energy demands for industry applications inform humanoid robot battery design choices at the cell level, pack level, and system level. Practical limits to current and next‐generation materials set optimization goals for battery design, where form factor, packaging, and auxiliary systems define the core of humanoid robots.

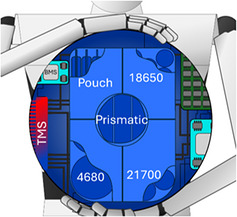

## 1 Introduction

Beyond mere simulacrums, humanoid robots are designed to mimic human behavior and appearance while proficiently replicating functions such as decision‐making and human interactions. Humanoid robots can possess superior advantages compared with other robot forms, not only due to their advanced equipment, including sensors and cameras, but also their ability to use tools and industrial environments evolved for human workers [1, 2, 3]. The ongoing fusion of artificial intelligence (AI) and humanoid robots further expands workforce capabilities. However, AI operations require significantly more electricity than typical internet searches.

As testaments of advanced engineering and technological capabilities, humanoid robots can fill roles in myriad dynamic scenarios including harsh and hostile environments for human workers [3, 4]. Their anthropomorphic design facilitates cohesion and conformity in human interactions, ensuring wide applications in healthcare, education, manufacturing, and disaster relief. However, executing complex tasks requires significant advances in technologies at the interface of mechanical engineering, materials science, electrical engineering, and computer science [2, 3, 4]. As engineers, scientists, and developers tackle mechatronic challenges of optimizing and integrating various systems, at least one key aspect of humanoid robots requires more attention: energy storage in the form of high‐performance batteries.

The needs and challenges of practical applications provide context for power solution requirements. Robotic technologies have matured over the last 40 years, often prioritizing advancement in demonstrable performance and task completion. While early pioneers sampled various power solutions, more recent work simply appropriates whatever commercial power solutions are available—most often: rechargeable batteries [5 ]. Recent demonstrations of prolonged operation reveal the demands in endurance that even current state‐of‐the‐art Li‐ion batteries struggle to accommodate [6]. Their size, bipedal mode of locomotion, and intended functions push the criteria for power source needs apart from those of consumer electronics or electric vehicles. Humanoid robots' constraints on power sources most notably include: size and shape of available volume, cost of transport for associated weight, and stability for consistent performance.

From energy density to discharge rates and safety, limitations of existing battery technologies pose significant hurdles to be addressed by “next‐generation batteries.” As humanoid robots evolve toward greater intelligence and utility, higher energy and power density batteries are necessary to support demanding semiconductor chips and sensors beyond actuators for mobility functions. Integrating humanoid robots into the workforce as steps in Industry 4.0 and 5.0 will improve productivity and safety for workers [2, 3, 4, 5, 7]. The gap between human metabolic efficiency and generalist robot energy efficiency means significant reserves of energy are needed to fill these labor‐intensive roles [8, 9, 10, 11]. The next generation of batteries will revolutionize power solutions beyond Li‐ion (graphite and layered lithium transition metal oxides such as LiCoO_2_, Li[Ni_x_Co_y_Mn/Al_1‐x‐y_]O_2_ (LCO/NCM/NCA) or lithium iron phosphate (LFP) electrodes with flammable electrolytes), but humanoid robots will benefit most from forward‐thinking design considerations to properly integrate high energy density batteries. Accommodate changes in form factors, implementing distributed hybrid energy systems, ensuring safe operation, and swapping modular battery packs will be essential. To assist in these tasks, this article offers an overview of imminent next‐generation battery technologies and their potential impact on humanoid robots.

**FIGURE 1:**
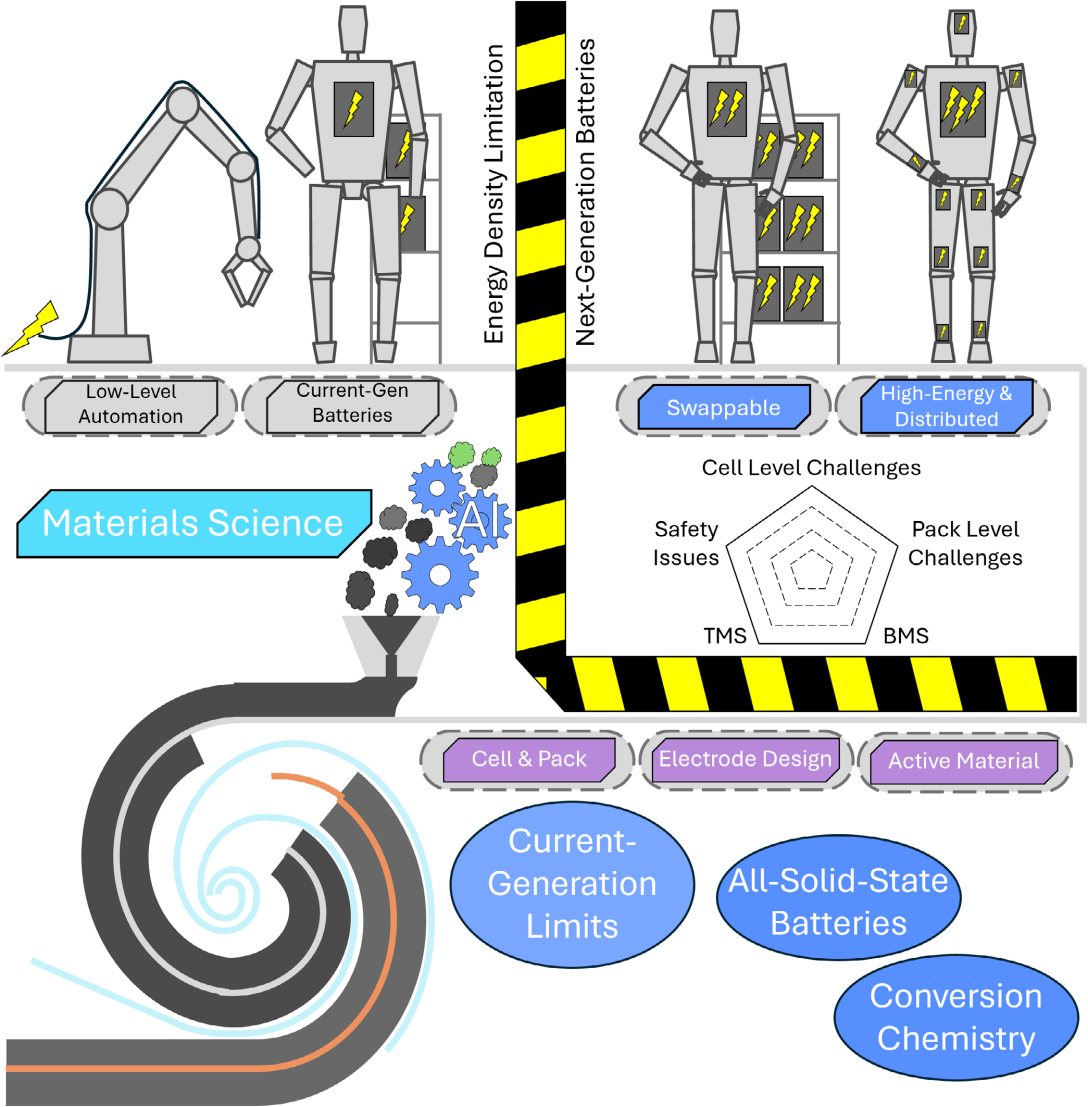
Barriers to a higher‐level automated workforce include several challenges realizing high energy density, next‐generation batteries. Next‐generation battery types require further breakthroughs via materials science, and cell and pack design re‐optimization for the new technologies.

Herein, historic and state‐of‐the‐art energy sources for various robotics contextualize current capabilities. Anticipated requirements given example applications are introduced and challenges specific to humanoid robot systems can be considered in energy system development. An overview of barriers discussed throughout are illustrated in Figure 1. The detailed limitations of current materials, prospective next‐generation battery technologies, and additional optimizations needed to realize a humanoid robot workforce compose the final section.

## 2 Requirements for Powering Humanoid Robotics

Humanoid robots present significant challenges in energy density, compared even to electric vehicles which have fueled progress in battery materials and manufacturing technology toward the goal of 1000+ km ranges. The shape and bipedal nature of humanoid robots emphasize particular power solutions that minimize mass—lowering the load on joints and motors—and sophisticated designs for integration into the system as a whole. Diverging approaches endeavor to balance stability, power, and energy density of viable mobile power sources. Designs of power sources vary greatly and are often single, monolithic packs that are either easily exchangeable to swap quickly or be permanently embedded in the torso, but may also develop toward several smaller packs distributed throughout the humanoid robot for mutual structural support. The energy–power–stability trade‐offs span material, cell, pack, and system level considerations, with interconnected technological design considerations to suit the application.

### 2.1 Introducing Energy Systems for Humanoid Robotics

**FIGURE 2:**
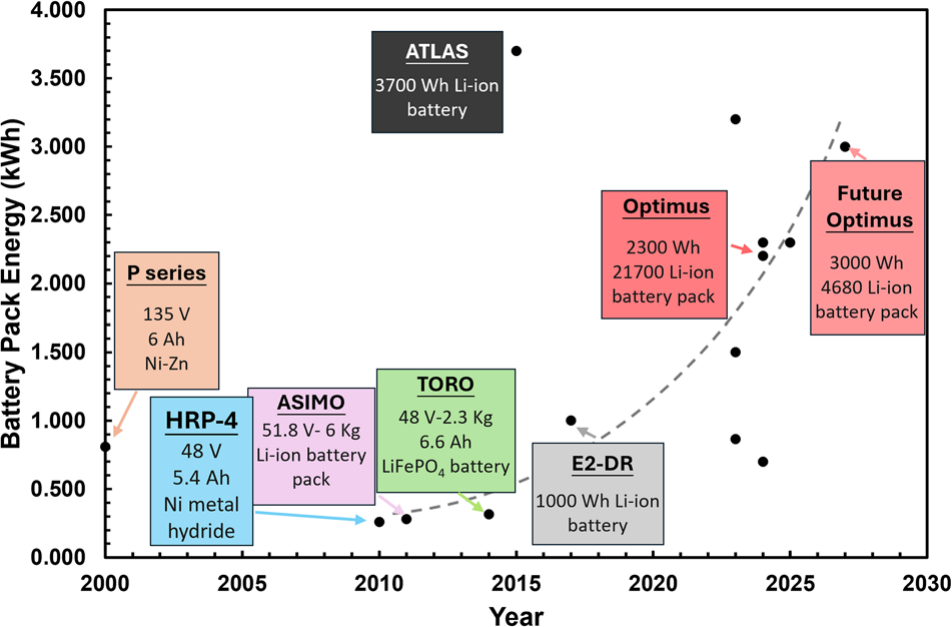
Advances in humanoid robot capabilities and battery pack progression. Li‐ion battery technology has become ubiquitous in humanoid robots, where a high energy capacity is most favored. Advances in AI and mechatronic technologies have grown exponentially in recent years, outpacing current generation battery materials.

Demand for humanoid robots in various industries is growing rapidly, spurred further by recent advances in AI capabilities and mechatronic technologies. As generalist robots, humanoid robots should be capable of performing complex tasks over extended durations with ever‐increasingly sophisticated robotic capabilities. Power and energy needs increase with their capabilities, up to 3 kWh today—see Figure 2. Humanoid robots are enabled by a range of power supply systems, lately by batteries of various form factors and chemistries [9]. Implementing appropriate power conversion systems, the power source(s) provide energy for all electronic systems, including sensors, processors, and motors [12]. Operational efficiency, capability to complete tasks, and mobility depend upon their sources and energy management. As such, electronic systems maximize efficiency by reducing losses to thermal waste and generators.

For humanoid robots, battery capacity (*Q* in Ah) is often considered with respect to material mass (*m*) for specific capacity (Ah ${\mathrm{kg}}^{-1}$) by material, cell, or pack and by volumetric (*V*) capacity (Ah $L^{-1}$). Starting at the material level, theoretical specific capacity follows from the number of charge carriers (*n* e.g., number of Li per formula unit) for a given unit mass (typically molar mass *M*, g mol^−1^) and Faraday's constant (*F*, 94850 C mol^−1^), converted from Coulomb × seconds to Ah, as in Equation (1).

(1) $$Q=\frac{nF}{3600\times M}$$

The product of capacity and the battery chemistry's average operating voltage (U in volts, V) gives specific energy (e) by Equation (2),

(2) $$e=C\times U\;\text{in Wh}\;{\text{kg}}^{-1}$$

and relates to energy density (w) by the material's density, (ρ, g cm^−3^ or equivalently kg L^−1^) per Equation (3).

(3) $$w=e\times \rho \;\text{in Wh}\;L^{-1}$$

At the material level, intrinsic limitations in its usable portion charge carrier lower the practical specific energy. Energy density are similarly scaled further by porosity and additives in cast electrodes. Cell‐level energy density/specific energy ($w_{c}$ and $e_{c}$) must account for active material (cathode, anode, electrolyte) and additional volume/mass of inactive components, including separator, current collectors, tabs, insulators, and casings. Efficiency of packing and linking cells together scales cell to pack level ($w_{p}$ and $e_{p}$). Finally, scaling to the system level ($W_{\mathrm{system}}$ and $E_{\mathrm{system}}$) factors in the Battery Management System (BMS), Thermal Management System (TMS), and other safety components, critical for integration and functionality.

(4) $$E_{system}\propto e\times c\times p\times s$$

For nearly all power sources available to humanoid robots, a similar set of parameters must be considered, as the weight of the battery or fuel adds to the encumbrance born by robot's joints and motors. The unitless Cost of Transport (CoT), following from energy expended ($E_{total}$) per unit distance traveled (d), total mass ($m_{total}$), and gravity (g) as in Equation (5)

(5) $$CoT=\frac{E_{expended}}{m_{total}dg}$$

is a meaningful metric for energy efficiency in mobility. The CoT of humanoid robots, as bipeds, is much more challenging to optimize compared to wheeled vehicles/robots. Improving (decreasing) CoT through high specific energy is extremely important for maximizing the energy available with minimal mass contribution. The CoT will be discussed further in Section 2.2.

Table 1 shows various power sources for humanoid robots; these sources are used as the sole energy system, or in combination for hybrid energy systems. Many battery technologies employed in the broad category of mobile robots have been previously reviewed [9, 13]. Some advantages, disadvantages, and use cases in humanoid robots are outlined in the following.

**TABLE 1:** Power Source Technologies' Advantages, Disadvantages, and Applications for Humanoid Robots.

Power source	Advantages	Disadvantages	Applications
Tethered	Unrestricted power	Mobility limitations	High‐power, stationary applications
Hydrogen fuel cell	Environmentally friendly Sustainable power High energy density	Expensive Low safety Complex fuel storage and delivery	Long‐duration tasks High‐energy applications
Nickel‐metal hydride battery	High safety Environmentally friendly	High self‐discharge rate Low energy density	Low‐power applications Low‐cost designs
Supercapacitor	Fast charge/discharge Long cycle life	High self‐discharge rate Low energy density	Hybrid energy systems Regenerative braking
Lithium‐ion battery	High energy density Long cycle life	Safety concerns Sensitive to temperature	High‐energy devices long‐time operation
Lithium‐polymer battery	High flexibility density Lightweight	Low ionic conductivity Sensitive to overcharge/discharge	
	Flexibility required design		
Solid‐state battery	High energy density Improved safety	Low power density and ionic conductivity Sensitive to temperature Early‐stage technology	Long‐endurance missions High‐energy applications
Lithium sulfur	Higher energy density than lithium‐ion battery Low price	Weak cycling stability Safety concerns Temperature sensitive	High‐energy applications
Primary battery	High energy density No maintenance Low cost	Single‐use Environmental impact	Emergency scenarios Critical, off‐the‐grid operation

#### 2.1.1 Tethered

Tethered robots benefit from unrestricted power consumption. Industrial spaces often are equipped with higher voltages of e.g., 480 V, 3ϕ power with sizable currents, enabling continuous execution or repetition of high‐power tasks, such as those executed by robotic arms for specialized tasks. External logic centers are readily enabled in these cases, as sensor and control signals can be routed through the tether, also. Additional converters (AC/DC) are required within the body or control module, however. The restrictions on dynamic or improvised movements due to the tether's limited reach and risk of entanglement inhibit the coherency and generalizability of humanoid robots.

#### 2.1.2 Fuel Cells

Fuel cell power systems are typically quiet, clean, and sustainable. Hydrogen fuel cells or direct methanol fuel cells (DMFC) provide competing technologies. Hydrogen fuel cells utilize a proton exchange membrane (PEM) that yield a product of only water but is restricted by hydrogen storage capabilities and refill infrastructure [14]. The DMFC produces water and CO_2_ but uses liquid methanol for a denser, easily refilled fuel source [15, 16]. Fuel cells are primarily reliable in steady power applications, however, so test beds like the HOAP 2 robot exemplify their ideal application in hybrid systems with batteries, which are better suited for peak power demands [17].

#### 2.1.3 Ni‐Metal Hydride (NiMH) Batteries

As an evolution of NiCd metal hydride batteries, the elimination of heavy metals has encouraged the use of NiMH batteries since the early 2000s. The HRP‐2, HRP‐4 and HOAP 2 robots utilized NiMH batteries at times [17, 18]. The low energy density and high self‐discharge rates generally inhibit practical application in humanoid robots.

#### 2.1.4 Supercapacitors

Supercapacitors are used for fast charge/discharge applications where short bursts of high power must be delivered. Their net energy storage is low compared to other power sources, however, and so are typically integrated into hybrid systems for humanoid robots, for example, high‐torque short‐duration needs. Supercapacitor technology can help supplement main power supply batteries [19].

#### 2.1.5 Lithium‐Ion Batteries

Rechargeable Li‐ion batteries dominate consumer electronics and electric vehicles, owing to high energy densities, low self‐discharge, and long system lives. Standard commercial Li‐ion batteries use graphite anodes, layered lithium‐metal‐oxide (LiMO_2_) or olivine LiFePO_4_ (LFP) cathodes, polymer separators, and organic liquid electrolytes [20, 21]. Layered oxide cathodes enable higher energy density, while LFP cathodes enable longer system life over repeated cycling. Organic liquid electrolytes are the source of combustible material, leading to safety concerns in the case of damage or fire [22]. Li‐ion batteries may use somewhat flexible pouch cells or hard cylinder and prismatic form factors dominant in electric vehicles.

#### 2.1.6 Lithium‐Polymer (LiPo) Batteries

Utilizing lithium‐polymer gel electrolytes [23], rather than liquid electrolytes, safety issues are somewhat lessened, and flexibility is enabled by the pouch form factor. Compared to other Li‐ion batteries, high energy density is achievable by thin electrolytes serving as a separator itself; however, they are more limited in charge/discharge rates and more sensitive to overcharging [24].

#### 2.1.7 Solid‐State Batteries (SSBs)

The next‐generation battery of choice for high energy densities, all‐solid‐state batteries with inorganic ceramic compounds or glassy electrolytes may also eliminate safety concerns of combustible electrolytes [25]. SSBs maximize active components of the batteries and potentially enable Li‐metal or alloying anodes for high energy density [26]. SSBs are still in development, with limited prototyping approaching true commercialization.

#### 2.1.8 Conversion Chemistry Batteries

Rather than intercalation chemistry typical to Li‐ion or LiPo batteries, more energetic chemical reactions may be leveraged in so‐called conversion chemistry batteries. Prominent examples include lithium–sulfur (Li‐S) [27] or lithium‐oxygen (Li‐O)—often as Li‐air batteries, designed to use oxygen from ambient air [28]. While some low specific energy metal‐air batteries, e.g., Fe‐air and Zn‐air have been commercialized, other conversion chemistry batteries are still immature, suffering from reaction kinetics and reversibility issues [29], but they may be key to high specific energy density applications (Li‐air and Al‐air). Implementation of batteries that breathe (Li‐air) will require unconventional designs for good battery health.

#### 2.1.9 Primary Batteries

Unlike rechargeable batteries, primary batteries provide a readily usable energy source in an environment without accessible charging infrastructure. Under disaster relief scenarios, sufficient supplies of primary batteries enable efficient operations without facing disruptions of charging time and access to charging infrastructure.

### 2.2 Current State‐of‐the‐Art Humanoid Robot Batteries

To test humanoid robots' state‐of‐the‐art locomotion performance on equal footing, a race was held in China in the Spring of 2025 [6]. A series of humanoid robots competed against one another in a half‐marathon, with human handlers running alongside them. An average human can be expected to attempt a half‐marathon at a pace of a 10‐min mile and so complete the 13.1 mi (or 21.1 km) in around 2 h 15 min, consuming 1200–1700 calories [30]. Running such a distance provides an analog of the repeated stresses and energy demands to operating under load for two hours or more. The caloric cost to humans translates to 1.4–2.0 kWh and provides a baseline requirement for the required energy a humanoid robot may require for such a demanding task. As reported in a recent review by Shi and Pikul [8], CoT of mobile robot power sources fall short of endurance benchmarks set by humans and animals. A survey of some state‐of‐the‐art humanoid robots' claimed battery capacity (up to 3 kWh), speed, and runtimes are compiled in Table 2. The ranges, projected from the prospective speed and battery runtime, are benchmarked against the length of half‐marathon. Few robots of today appear capable of meeting the challenge with these generous interpretations.

**TABLE 2:** Battery Pack Capabilities of Several State‐of‐the‐Art Humanoid Robots.

Name	Manufacturer	Type	Energy [kWh]	Speed [km ${\mathrm{hr}}^{-1}]$	t_run_ [hr]	Range [km]^1^	% of half‐marathon^1^	Hot swap
Optimus	Tesla	Li‐ion (NCM)	2.3	8	1.5	12	57%	N
Atlas	Boston Robotics	Li‐ion (custom)	3.8	9	1	9	43%	N
Figure 01	Figure AI	Li‐ion (NCM)	4.32	8	2	8.64	41%	N
Figure F.02	Figure AI	^2^	2.25	4.32	5	21.6	102%	^2^
G1	Unitree	^2^	^2^	^2^	10	34	166%	^2^
H1	Unitree	Li‐ion (LFP)	0.864	12.24	2.5	30.6	134%	Y
Apollo A1	Apptronik	Li‐ion (NCM)	3.2	3.6	4	14.4	68%	Y
A2 Ultra	Agibot	Li‐ion (^2^)	0.7	7	1.5	10.5	50%	Y
Digit	Agilty	^2^	^2^	5.4	4	21.6	102%	^2^
GR‐1	Fourier	Li‐ion (^2^)	^2^	18	^2^	^2^	^2^	^2^
NEO	1x Technologies	^2^	^2^	10.8	2	21.6	102%	^2^
Tiangong Ultra	UB Tech	^2^	^2^	10	1	10	47%	Y
ASIMO 2011	Honda	Li‐ion (^2^)	0.280	9	1	9	43%	^2^
Walker X	UB Tech	Li‐ion (^2^)	0.546	3	2	6	28%	^2^
TALOS	Pal Robotics	^2^	1.11	3	1.5	4.5	21%	^2^

^1^ Assuming reported Speed and t_run_ times are mutually accurate

^2^ Not publicly available

Ultimately, the winner of the robotic leg of Beijing 2025 E‐Town Half Marathon—finishing in 2 h and 40 min—was the Tiangong Ultra. This victory was enabled by three battery swaps, exemplifying the limitations of the commercial battery packs currently implemented in many humanoid robots. Where the Tiangong Ultra's practical range would be estimated closer to 6.3 km between hot swap, a Guiness world record for a humanoid robot was set by the Agibot A2, walking 106 km nonstop from night of November 10 through the morning of November 13, 2025 [31]. Similarly assuming hot swaps every 1.4–3 h, its range may have been an average of approximately 5 km. The 2026 Half Marathon was exhibited a robot named “Lightning” completing the course in 50 min 26 s, beating the human world record time by several minutes. This performance exemplifies both the capabilities of specialty robotics and—considering its impact with a barricade and fall after completion—the importance of live battery safety monitoring in real‐world scenarios.

Although humanoid robots and battery technologies have generally matured in parallel over the last 40 years, it is crucial to understand the state‐of‐the‐art in Li‐ion batteries—the leader in energy density. Developments have yielded consistent improvement in battery technologies, a culmination of material, cell, pack, and system level advances. It is notable however that since the advent of the Li‐ion battery with LiCoO_2_ cathode and graphite anode (both van der Waals materials) in 1991, for which Akira Yoshino, M. Stanley Whittingham, and John B. Goodenough won the 2019 Nobel Prize in Chemistry [32, 33, 34], Li‐ion batteries have remained fundamentally the same [35, 36, 37, 38].

For many mobile electronics, batteries have three basic requirements: high cell voltage, high specific capacity/charge density, and high reversibility. Battery materials and chemistry dominantly dictate these criteria. Operating voltage follows from the relative potential at which cathodic and anodic electrochemical reactions take place. High capacities are derived from specific charge and charge densities, which follow from the number of charge carriers per mass and unit volume of the materials involved. Highly reversible electrochemical reactions ensure performance does not degrade over hundreds or thousands of cycles. Material level advances in the form of compositional and morphological material design of layered lithium metal oxide cathodes has led to nickel‐rich NCM (Ni > 60% of transition metal composition e.g., NCM811 being 80% Ni, 10%Co, 10%Mn) as a recognizable state‐of‐the‐art cathode for Li‐ion batteries, and fundamentally similar to Sony's original 1991 rechargeable Li‐ion battery [32, 38]. Only recently has graphite been challenged by silicon/graphite composite anodes as a means of increasing energy density, at a cost of other capabilities. In contrast, LFP LiFePO_4_ cathodes sacrifice energy density for cycle life.

### 2.3 Requirements: Future Goals for Humanoid Robot Batteries

Given the above state‐of‐the‐art in lithium‐ion batteries, we revisit here the goals, challenges, and benchmarks for humanoid robots. There are many potential use cases of humanoid robots with various requirements. Common demonstrations include walking, running, or feats of agility like dances or backflips—all reliant on balance. Even simply standing requires active correction schemes for bipedal robots to maintain balance. For demonstrations beyond these basic tasks and closed‐course scenarios, available power becomes critically important. Additional exertion of lifting and moving heavy loads—as in a warehouse, manufacturing, healthcare, or rescue work settings—requires higher and sustained torques which increase power demands, as well as heating joints and motors.

The practical use of humanoid robotics to supplement workforces and conduct such backbreaking work is an appealing application that has funded many robotics projects. Hyundai Motor Group acquired a majority stake in Boston Dynamics to develop the Atlas humanoid robot to fill roles in their manufacturing plants [39]. The demanding and dynamic workload for moving heavy objects such as panels, welding equipment, or batteries limits real runtime of onboard batteries. MBW reported their Figure F.02 robots working in manufacturing lines over 11 months by exchanging battery packs throughout the 10‐h shifts [40]. Additional considerations are cooling systems required to mitigate thermal buildup on high‐torque joints or the batteries themselves, as well as computational processes to perceive their surroundings, maintain balance, and follow the requisite logistics of their tasks.

**FIGURE 3:**
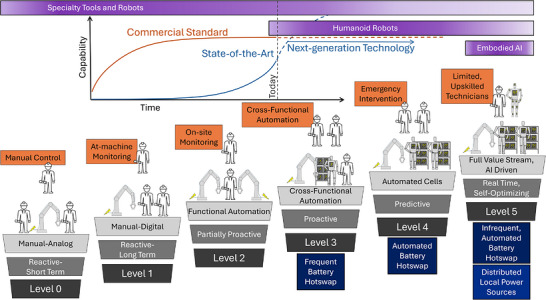
On the roadmap to manufacturing automation, integrated humanoid robots with M2M communication can lower risk to workers. Next‐generation technology in mechatronics, AI, and batteries will be essential to Industry 4.0 and beyond.

Current autonomous systems, such as those used in industry, require close human supervision by operators for safety and handling disruptions. Five levels of manufacturing automation have been outlined on a roadmap toward Industry 5.0—see Figure 3 [7]. At the first level of automation, collaborative robots (cobots) use direct at‐machine monitoring and control and so are minimally relevant to humanoid robots. Levels 2 to 3 increase machine‐to‐machine (M2M) communication and capabilities, enabling Industry 4.0 tier operation, in which each human operator may supervise many automated tools and potentially humanoid robots, with proactive rather than reactive automation [4]. Labor‐intensive jobs in agriculture, construction, industry, and transportation demand metabolic energy consumption (power) rates in the range of 2–6 kcal 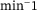, or 140–420 W, some rare instances as high as 12.8 kcal 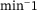 (890 W) and approximately 1 kcal 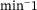 at rest [11]. Supposing duty cycles of 66% (e.g., 10 min active and 5 min rests) and a conservative 100 W rest power, an 8‐h shift requires at least 1 kWh and more likely upward of 3.0 kWh.

Though many humanoid robot specifications indicate over 3 kWh battery capacity, the Beijing 2025 E‐Town half‐marathon exhibited current battery systems limitations to run times under operational loads to a couple of hours at best, often with 1–5 h to recharge [8]. The discrepancy between biological efficiency of humans and the mechatronic efficiency of generalist humanoid robots limits comparable energy use to ∼1/4th the requirement for a full shift. A human supervisor's 8‐h shift would then require at least two robots or excess swappable batteries for every role, such that one is charging while its counterpart is active, a so‐called fleet approach [9, 29, 40]. Removing this redundant workforce requirement necessitates a fourfold increase in battery capacity to 10–20 kWh or high mechatronic efficiency [41].

## 3 Challenges of Batteries Specific to Humanoid Robots

The prospective adaptability of humanoid robots for dynamic environments and tasks also set challenging bounds on power sources. Restrictions in employed battery technologies include power density and weight distribution, safety concerns, battery life, system life, fast charging capabilities, thermal management systems (TMS), and battery management systems (BMS). Synergistic advances of the battery pack, cell, system, and material technologies will be necessary to meet and exceed benchmarks with their respective domains of impact indicated in Table 3.

**TABLE 3:** The Challenges Specific to Humanoid Robots Assigned as Most Impacted by Material, Cell, Pack, and System‐Level Design Optimization.

Material design	Cell design	Pack design	System Design
Battery Life	Battery Life	Structural Integrity or Hot‐Swappable	System Life
Specific Capacity (Ah ${\mathrm{kg}}^{-1}$)	Energy Density (Wh $L^{-1}$ or Wh ${\mathrm{kg}}^{-1}$)	Capacity (Ah)	Weight Distribution
Battery Potential (V)	Cost	System Voltage (V)	Battery Management System (BMS)
Safety	Power Density (W $L^{-1}$ or W ${\mathrm{kg}}^{-1}$)	Power Density (W $L^{-1}$ or W ${\mathrm{kg}}^{-1}$)	Thermal Management System (TMS)

### 3.1 Cell Level Challenges

The energy storage system in humanoid robots must simultaneously satisfy three conflicting demands: high energy density for extended operation time, high power capability to handle dynamic actuation events such as walking and manipulation, and long cycle life under repeated charge/discharge cycles with transient loading. It is worthwhile noting that for certain class of applications, these demands would have different priorities: e.g., humanoid robots for disaster relief operations (e.g., rescuing humans trapped in a burning building, searching a flooded or collapsed structure, etc.) would have much lower priority for cycle life performance compared to operation time and power capacity. Note that 3 kWh battery would cost ca. $300 at the current cost of $100 ${\mathrm{kWh}}^{-1}$, representing a very small portion of the overall cost of the unit operating under such harsh environments. Under such circumstances, even non‐rechargeable batteries with superior energy and power capacities would be much better suited.

Cylindrical, pouch, and prismatic cell form factors are commercially established, meeting these requirements with distinct trade‐offs [42]. The 18 650 and 21 700 cylindrical cells are favored for their mechanical robustness, thermal stability, and manufacturing maturity. Tesla Optimus previously incorporated LG INR21700‐M50LT cylindrical cells composed of 126 cylindrical 21 700 cells, arranged into two independent modules connected in series. Each module contains 63 cells, and the total energy is approximately 2.22 kWh, supporting the platform's multi‐joint locomotion, manipulation, and onboard computation during high‐load tasks.

In contrast, pouch cells offer a lightweight, flexible form factor with superior volumetric energy density, making them highly adaptable to curved or compact regions within the humanoid body. However, their reliance on external compression structures and susceptibility to swelling or thermal degradation necessitates additional protective measures at the system level [43]. Prismatic hard‐case cells provide a flat, stackable design that simplifies module integration, but they are generally bulkier and heavier per unit energy compared to pouch cells, and their rigid casing can hinder seamless integration in space‐constrained or articulated zones [42].

Beyond the form factor, comparative cost modeling on a large production scale reveals further trade‐offs. 18 650 and 21 700 cylindrical cells achieved moderate production costs due to mature, high‐yield manufacturing infrastructure. Despite having the lowest material costs, pouch cells pose significant challenges for automated high‐throughput production, particularly in achieving consistent quality and yield, and prismatic cells often are even more challenging to develop and optimize but offer structural rigidity that may contribute to system design. Particular next‐generation chemistries and physical requirements will be more compatible with these form factors or emerging designs [44].

Humanoid robots often exhibit intermittent but high‐amplitude current profiles, especially during walking, standing up, or performing object interactions, which induce accelerated aging at the cell level. These considerations are further modulated by the choice of active materials, which affect both energy density and cost, ultimately influencing cell selection for specific humanoid robot design priorities. To meet the long‐term performance needs of humanoid robots, future battery cells must be co‐optimized for energy–power–stability balance, with emphasis on electrode engineering, electrolyte additives for high‐rate stability, and in situ diagnostics tailored to dynamic robotic loads.

### 3.2 Pack Level Challenges

To maximize spatial efficiency and mass distribution within humanoid robots, battery packs are increasingly being structurally embedded within the torso, transforming them from standalone energy reservoirs into mechanically integrated subsystems [45]. In this architecture, the torso functions not only as a protective housing for high‐energy cells but also as a structural backbone that supports mechanical loads and provides routing channels for power and data. By co‐locating energy storage with the robot's central support frame, this approach minimizes power transmission distances, reduces electrical losses, and simplifies internal cabling, thereby enhancing both the mechanical and electrical coherence of the overall system.

Alternatively, Apollo A1 utilizes a LIB system rated at 480 Wh and 51.8 V, delivering up to 1080 W of peak power. It features a hot‐swappable design enabling battery replacement in under 30 s and incorporates a custom power distribution system to support real‐time reconfiguration and circuit protection. Each Apollo A1 battery pack supports up to 4 h of continuous operation under typical working loads [46], enabling extended task execution without immediate recharge requirements. Walker S2 is a new industrial humanoid robot capable of autonomously removing and swapping its own batteries. In a demonstration released by the manufacturer, the robot approaches a charging dock, rotates, and ejects one of its rear‐mounted batteries into an empty slot while simultaneously inserting a fully charged replacement [47]. This operation is enabled by a dual hot‐swappable battery configuration, ensuring continuous power during the exchange process, which implies a move toward standardized battery interfaces and pack formats that facilitate automated exchange, interoperability, and uninterrupted operation—features critical to scalable deployment.

Looking ahead, emerging battery concepts may further improve integration and form factor efficiency. Dahiya et al. [48] explored micro‐battery arrays and structural batteries, particularly in small‐scale or soft robotic platforms, but these technologies remain at early stages in humanoid‐scale applications due to trade‐offs in areal energy density and manufacturability. As robotic platforms increasingly adopt distributed sensing, actuation, and computation, these limitations highlight the need to rethink power delivery paradigms in favor of more modular and resilient energy architectures. By allocating energy storage and management locally to individual modules or joints, these systems enhance the autonomy of each functional unit, enabling fine‐grained control and localized actuation even in the presence of partial system failures. Such decentralization also improves fault tolerance by minimizing single points of failure and facilitating controlled degradation.

### 3.3 Safety Issues of Humanoid Robotics

Humanoid robots often operate close to humans and under variable mechanical stress. Therefore, safety is another pressing issue. Particularly Ni‐rich NCM based Li‐ion batteries pose risks due to flammable electrolytes and instability electrode materials, raising concerns about thermal runaway and toxic leakage [49, 50]. To address safety concerns, recent research has focused on safer chemistries and material‐level improvements, such as the use of non‐flammable gel or solid polymer electrolytes, corrosion‐resistant metal anodes, and passivation‐suppressing interfacial coatings. These advances help suppress dendrite formation, reduce internal short‐circuit risks, and enhance thermal stability, supporting the development of intrinsically safer battery systems for deformable and mobile robotic platforms [28].

Multiple levels of robotics operations require separate considerations. At the cell level, positive temperature coefficient (PTC) thermistors, current interrupt devices (CIDs), safety vents, and protection circuitry are specialized safety devices. At the pack level, passive structural reinforcements and thermally absorbent materials (e.g., phase change materials, PCMs) are integrated to prevent the propagation of thermal runaway. Complementing these hardware safeguards, advanced BMS implementations support fault prediction, thermal runaway preemption, and system‐level shutdown through real‐time coordination with the robot's main controller. At the system level, predictive load scheduling and fault‐tolerant power zoning improve operational robustness, enabling robots to maintain degraded functionality under partial pack failure [51].

In addition to preventive strategies, emerging efforts should also focus on real‐time fault sensing and post‐failure containment to establish multi‐layered protection for safe robotic operation. Redundant battery modules should be configured with careful consideration of both spatial constraints and power distribution balance, ensuring continued functionality in mission‐critical scenarios such as disaster relief operations. Operational environments for humanoid robots may include significant vibrations and impacts, such as by falls, which may damage battery packs or circuitry. Rigorous fabrication specifications and impact testing proposed for humanoid robot circuitry [52] should extend to battery pack design, as catastrophic failure of Li‐ion batteries poses a severe hazard to surrounding humans, other humanoid robots, and infrastructure.

### 3.4 Thermal Management of Humanoid Robotics

Typical current‐generation Li‐ion batteries are optimized for peak performance and longevity in operating temperatures of 15∘C–35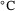. Low‐temperature (∼0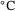) operation risk failure at kinetically limited steps (proper Li‐mobility), implementing heat pumps to ensure proper temperature during charging or operating. High temperatures (∼60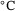) gives rise to capacity loss through side reactions and gas evolution. Subsequent thermal runaway is a serious threat, requiring strict engineering to mitigate [49, 50, 53, 54]. Evolution of gases by degradation of flammable electrolytes and cathode materials can produce a recipe for runaway hazardous chemical reactions within a cell. The released energy can heat nearby cells, perpetuating a dangerous chain reaction. As such, thermal management systems monitor and actively maintain operating temperatures to facilitate healthy battery operation and preempt thermal runaway. The operation of these pumps, fans, and monitoring systems introduce inherent energy overhead.

Conventional centralized power architectures, while prevalent in current robotic systems, impose significant limitations as robots grow in complexity, autonomy, and modularity [48]. Concentrating energy storage in a single, monolithic pack often leads to thermal management challenges, as localized heat accumulation in densely integrated enclosures can compromise both performance and safety.

Ardebili et al. [55] presents a comprehensive modeling and validation framework tailored to the lithium‐ion power system used in NASA's Robonaut 2 humanoid robot. They developed a multi‐scale thermo–electrochemical model, which ranges from individual cells to the full 300‐cell pack. The simulation predicts spatial temperature gradients (–40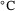 to 60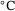), transient heating behavior, and inter‐cell thermal imbalances, and is validated against experimental discharge data under multiple C‐rates (0.5 C to 2 C). Despite its technical rigor, the analysis focused on steady‐state and moderate transient profiles but does not fully resolve rapid thermal spikes that could occur during unexpected load surges or thermal runaway initiation. Moreover, battery aging effects such as increased thermal resistance and cell impedance were not included, despite their critical role in long‐term performance and thermal reliability.

This omission underscores the need to integrate accurate state‐of‐charge (SOC) and state‐of‐health (SOH) estimation into thermal modeling frameworks. In particular, the conventional battery aging models developed for electric vehicles or stationary storage systems fail to capture the non‐uniform, burst‐intensive load profiles characteristic of humanoid robotics. As a result, lifetime prediction and pack sizing remain highly uncertain without adaptive, real‐time SOC/SOH tracking mechanisms. Moreover, centralized wiring schemes introduce spatial and mechanical constraints, complicating routing in articulated or soft‐bodied structures.

To further advance large‐scale distributed battery monitoring, Huang et al. [56] proposed the concept of the Internet of Batteries (IoB), which uses a wireless battery management system to achieve continuous remote health tracking. This approach enhances the flexibility and scalability of the system while significantly reducing the complexity and weight brought by traditional wired connections.

Through monitoring battery thermal behaviors and managing battery health to mitigate deterioration or thermal runaway, battery thermal management systems effectively counter heat build‐up during operation, particularly stints of high power such as fast charging [49]. Some thermal management systems may pair passive heat transfer mechanisms (e.g., heat pipe cooling or PCMs) with the above monitoring to control battery charge/discharge rates to maintain optimal operation. More active thermal management systems apply air (natural or forced convection), liquid (direct, indirect, cold‐plate, or channel schemes), or thermoelectric materials [53]. Integration of both active and passive components for hybrid systems strive for extra efficiency and safety from thermal runaway prevention through synergistic redundancy, at the cost of complexity.

For humanoid robot applications, active heating and cooling systems introduce notable design challenges. Swappable and modular battery packs must include thermal management designs, with a means of integrating with the humanoid robot systems for cooling/heating and thermal runaway mitigation. Structurally embedded packs enable more robust active thermal management via configuring air or liquid cooling channel with fans or pump with a system's heat pumps or fresh air source (passive air cooling) [53]. Liquid cooling provides more compact solutions but greater mass, while air cooling requires greater volume—requiring cells be less tightly packed for sufficient airflow. The additional mass and volume of the TMS should be considered at the system level specific energy and energy density. As such, less thermally stable materials that require more strict thermal management often suffer greater losses at the pack‐to‐system level, and non‐flammable solid electrolytes offer means of lessening thermal management needs, improving system level energy densities.

### 3.5 Battery Management Systems of Humanoid Robotics

Traditional battery management systems (BMS) primarily focus on core metrics such as SOC, SOH, state of power (SOP), remaining useful life (RUL), and energy efficiency (EE). These indicators, while effective in applications such as grid storage and electric vehicles, are insufficiently adaptable for task‐driven robotic systems.

In humanoid robotics, the battery must operate under diverse and dynamic conditions, including extreme temperatures, humidity, high pressure, and underwater environments that conventional BMS architectures struggle to handle in real time. Zou et al. [57] proposed a paradigm shift from battery‐centric to task‐centric battery management, integrating task characteristics, including type, priority, and time constraints with battery state prediction to enable dynamic task adjustment and intelligent energy scheduling. This task‐aware approach calls for a BMS that is co‐optimized with task planning, state estimation, and artificial intelligence algorithms, making it particularly suited for complex, adaptive, and multitasking robotic operations.

Therefore, as energy systems in humanoid robots become increasingly integrated with control and actuation subsystems, battery packs are evolving from stand‐alone storage modules to embedded, responsive components within the broader robotic architecture. This integration unlocks new capabilities, such as real‐time power reallocation based on task urgency, predictive energy budgeting across concurrent activities, and adaptive load balancing during mission execution. Such predictive, rather than reactive or merely proactive automation, will require comprehensive data on activity, performance, and environmental parameters to best develop and train algorithms. As a result, humanoid robots can achieve improved operational efficiency, resilience, and autonomy, especially in mission‐critical or resource‐constrained scenarios.

Wang et al. [58] systematically investigated semi‐open queuing network models to evaluate how battery charging technologies (fast vs. slow charging), charging policies (priority vs. dedicated), and charger quantity affect operational performance. Contrary to conventional assumptions, it revealed that slow charging can outperform fast charging after a certain number of cycles due to reduced degradation rates. Moreover, high‐priority tasks being charged first proved more cost‐effective than static and dedicated schemes. However, the absence of standardized battery formats, regarding pack size, voltage levels, connectors, and communication protocols remains a barrier to cross‐platform compatibility and modular system design. Drawing lessons from the UAV (unmanned aerial vehicle) industry, where pack standardization has catalyzed interoperability and scalable manufacturing, establishing similar standards for humanoid robotics may be key to unlocking broader ecosystem development and supply chain resilience [59].

## 4 Meeting the Needs of Humanoid Robotics

As cell, pack, and battery management solutions advance system life and safety, the energy and power densities are ultimately limited by the materials used. The range of electrochemical stability for cathode, anode, and electrolyte dictate the total usable capacity and window of operating voltage. Mass and charge transfer dynamics similarly determine the sustainable rate of charge/discharge. As outlined above, various energy technologies have been leveraged in humanoid robots over decades of development and for particular use cases. As Li‐ion battery technology has become a ubiquitous mobile energy solution, the commercialized conventional Li‐ion battery has evolved with the state‐of‐the‐art through persistent research and development. To meet the expanding energy needs of humanoid robots to achieve 10+ h of work, specific energy becomes a pivotal criteria in assessing next‐generation battery technologies. Some developing candidates in emerging battery technologies are highlighted here, with consideration of their key trade‐offs in the energy–power–stability balance at the system level.

### 4.1 Theoretical vs. Practical

**FIGURE 4:**
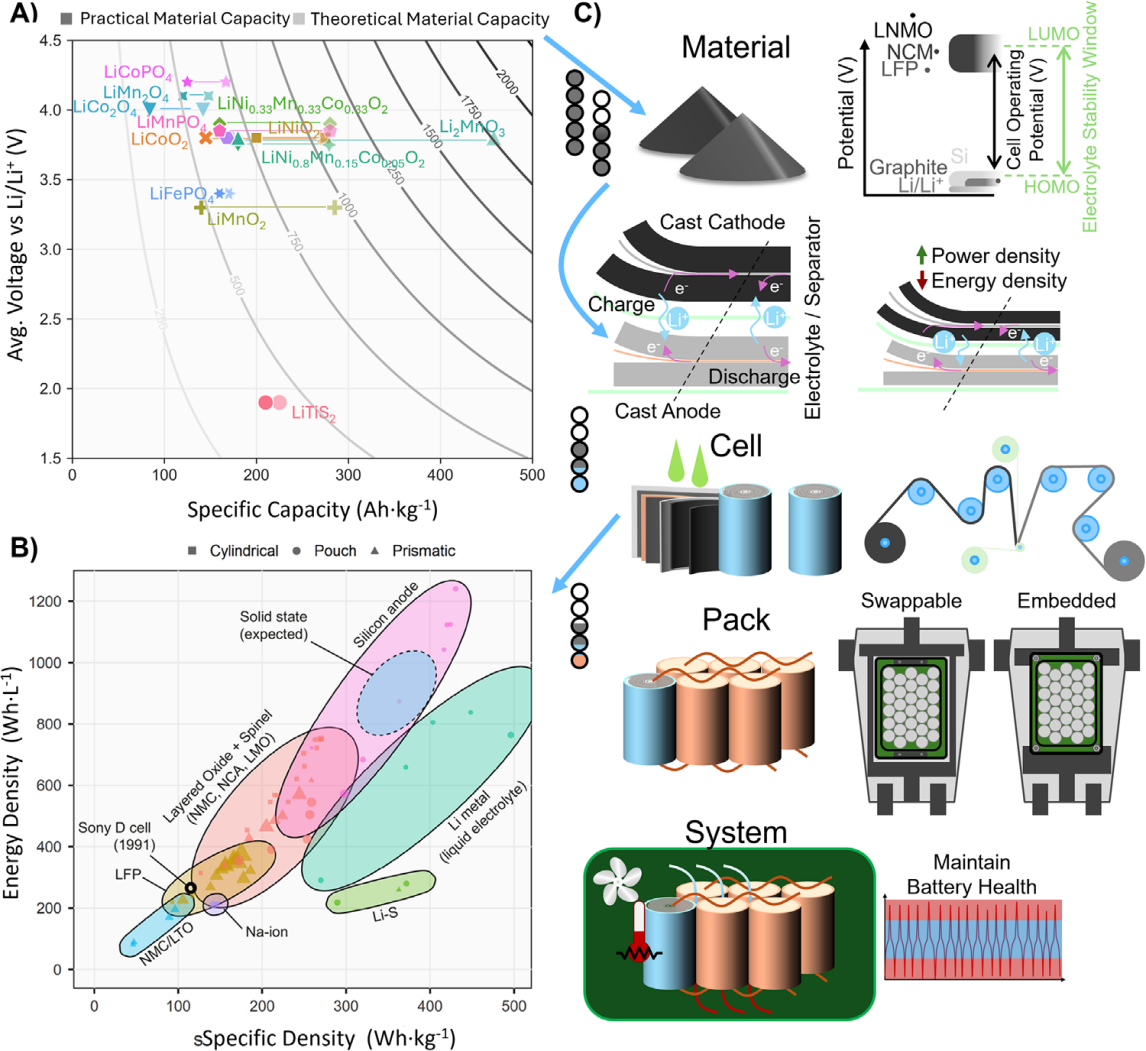
Battery, cell, pack, and system scaling. The (A) material operating potential and capacities—theoretical and practical—compared to (B) cell level specific density and energy density in common cylindrical, pouch, and prismatic formats. The data of had been compiled by Frith et al. [20] and reproduced with permission under the Creative Commons 4.0 license (http://creativecommons.org/licenses/by/4.0/). Final performance must consider attributes (C) including the material choices for specific capacity and electrochemical potential, electrode design and cell format, to then be integrated with pack features, such as swappable or embedded modules and system‐level safety and battery health systems: BMS, TMS, CIDs. Example scaling of usable energy density suffers losses in sequence of: material theoretical to material practical (white), cell‐level (grey), cell‐to‐pack (blue), and pack‐to‐system (orange) bubble shading.

From the inception of the rechargeable Li‐ion battery with pack‐level 100–120 Wh ${\mathrm{kg}}^{-1}$ in 1991, improvements to cell, pack, and system design have undoubtedly contributed to the increase in specific energy density up to 300 Wh ${\mathrm{kg}}^{-1}$ today [20, 42, 60]. Battery materials have improved alongside cell design, narrowing the gaps between theoretical and practical capacities shown in Figure 4A; however, there are ultimate limitations to beware of. Although the remaining capacity is an enticing asset to unlock, various mechanisms and phenomena present severe limits to overcome [61]. In a majority of current‐generation battery materials, where graphite has been a ubiquitous anode, improving electrochemistry and capacity has historically relied on improving the cathode active material (CAM) and electrolytes to minimize CAM degradation.

Further quantification of cell‐level volumetric energy density and specific density in Figure 4B, reported by Frith et al. [20], reveals the limit of current generation batteries, as they have progressed since the 1991 Sony D Cell. Alternative anodes to graphite have drastic effects, such as lower metrics for LTO anodes, or much higher specific density for silicon and Li metal anodes. Attributes such as those shown in Figure 4C consider material, cell, pack, and system design, which each compound contributions to the final performance. Accounting for material energy density (w), the portion of active components in the cell (*c*), and packing efficiency of the pack (p) for a number of cells ($n_{c}$), and integration into the system (s) yields a system's energy proportional to the cumulative product, as in Equation (3).

(6) $$E_{system}\propto w\times c\times p\left(n_{c}\right)\times s$$

Figure 4A highlights various current‐generation cathode materials' specific capacities and average operating voltage, the products of which yields specific energy (e). Relating energy density to specific energy beyond material level considers anode/cathode tap density ($\rho_{tap}$), ∼2 g cm^−3^, electrolyte and polymer separator/insulator density ∼1 g cm^−3^, and metal components such as cylinder cell can or pouch cell laminated aluminum, tabs, and current collectors ($\rho_{steel}∼$8 g cm^−3^, $\rho_{Cu}∼\rho_{Ni}∼$8.9 g cm^−3^, $\rho_{Al}∼$2.7 g cm^−3^), and finally empty space, plastic packaging, or control circuitry. A benchmark energy capacity can be derived from 10 kWh per pack of approximately 10 L (∼11.8″×7.3″×6.3″), which may fit in the torso of many humanoid robots and contain 60 (5×6×2) 4680 cylinder cells. Optimal hexagonal packing of 91% trends toward 74% but for the number of cylinder cells, $n_{c}$, in these pack volumes, tends to be near 80%, whether 18 650, 21 700, or 18 650). Including space for terminals, BMS, TMS, and safety components to yield battery system level energy density, cell‐to‐pack ($p(n_{c})$) × pack‐to‐system (s) integration may be 60% or as little as 33% efficient. As indicated in Figure 4B, the upper bound of solid‐state battery cells are anticipated to achieve this 1 Wh L^−1^ at the cell level only. A 2025 Global Market report ambitiously projects a 3× increase in energy density by 2031, reducing the current humanoid robot packs in the range of 3–7 L and 9–14 kg to less than 5 L [5]. Achieving this leap will rely on enabling solid‐state, silicon anode, Li‐S, and/or Li‐metal technologies at a commercial scale, reshaping preferential form factors to do so. The following discussion touches on the technical and material challenges to be overcome to possibly accomplish that goal.

#### 4.1.1 Bounds of Current‐Generation Battery Materials

Compared to legacy rechargeable Li‐ion batteries e.g., utilizing LiCoO_2_ cathodes and graphite anodes, current‐generation and next‐generation materials seek to optimize for particular application, with trade‐offs between stability, specific energy (Wh kg^−1^)/energy density (Wh kg^−1^), and cost of manufacturing.

Efforts to improve the accessible capacity of Ni‐rich layered lithium transition metal oxides present a challenge of stability. The cycling stability suffers drastically when utilizing a greater portion of Li than ca. 75% (∼4.3 V vs. Li/${\mathrm{Li}}^{+}$), preventing complete application of the theoretical capacity and voltage range. Air stability also decreases for these high‐Ni materials, where simultaneous exposure to H_2_O and CO_2_ degrades the cathode material, thus requiring synthesis and handling in dry environments, such as dry rooms with < 1% relative humidity (RH). Dopants, structural engineering of synthesized cathode crystals, and coatings each may provide further, incremental improvements to the conventional Li‐ion battery. NCM and NCA materials of various metal compositions tune energy density, thermal and mechanical stability, and cost. Synergistic properties of Co and Mn or Al largely dictate crystalline stability during Ni redox. Maximizing the redox‐active Ni and corresponding use of Li in the layered oxide, ultra‐Ni‐rich NCM pushes toward the theoretical capacity of ∼274 Ah ${\mathrm{kg}}^{-1}$ of cathode active material while reducing cost and ethical sourcing of Co, but lessens the stabilizing effects of the other cations. Particularly, NCA (typically 80% or 90% Ni) earned early adoption in EVs through high energy density, specific power, and cycle retention—the lower thermal instability at high charge states necessitates robust thermal management, however. For an average operating voltage of nominally 3.7 V, NCM cathodes could theoretically achieve ∼1000 Wh kg\rlap^−1^_CAM_.

As a mature anode material, commercial graphite has little room to improve, already exhibiting high capacity (372 Ah kg^−1^) and cycle retention. Though more expensive than natural graphite, current research and development tends to focus on graphite synthesis from various precursor sources (tar pitch, needle‐coke, petroleum‐coke, etc.), toward purity, crystallinity, morphology, and coatings for optimizing parameters including electronic conductivity, fast charging capabilities, and thermal stability.

The olivine LFP cathode operates at lower average voltage (∼3.2 V) than layered oxides (∼3.7 V), but with excellent reversibility owing to minimal volume change and good stability. The lower ionic and electronic conductivity of LFP (1D Li^+^ conduction pathways) compared to NCM materials (2D conduction) do limit the charge rates, and thus power. To compensate, olivine cathodes use nano‐scale particles with carbon coating [62]. As a polyanion compound, the specific capacity is lower and ion transfer kinetics slower. Less stringent thermal management due to better thermal stability and lower specific power improves the cell‐to‐pack conversion over batteries with NCM cathodes. Degradation by powder surface oxidation requires that synthesis/handling of the powder, and cast cathode still largely be done in controlled environments i.e., < 1% RH [63]. Nevertheless, the lower cost of Fe and phosphates components improve relative cost, overall yielding increased favoring of LFP over NCM chemistries where specific energy and power are not prioritized.

Si and SiO_x_ anodes offer much greater theoretical capacity than graphite but suffer drastic volume expansion (up to 400%) as they alloy with Li during charge. Similar to graphite, the crystallographic anisotropy of the volume expansion during Li incorporation encourages casting of randomly oriented powders, nanostructured anodes, or amorphous materials [64]. Strategies to improve Si use in silicon–carbon composite anodes enable significant improvements to capacity [65]. Such composite electrode recipes can typically be integrated into existing electrode fabrication methods.

These current‐generation electrode materials emphasize trade‐offs in stability (LFP, graphite), specific energy (Ni‐rich NCM, Si), and manufacturing cost. Where LFP sacrifices specific energy for stability, Ni‐rich layered oxides sacrifice stability for material‐level specific energy (w). Including losses at the cell (c), pack (p), and system (s) level components, Frith et al. [20] posit that LFP||Graphite and NCM||Graphite‐SiO_x_ packs sometimes yield similar final energy density (Wh kg^−1^, despite NCM||Graphite‐SiO_x_ exhibiting much higher theoretical capacities, owing to its lower thermal stability requiring additional TMS components—lowering system level efficiency (s) in Equation (6).

### 4.2 Next‐Generation Battery Materials

Improving battery storage capabilities has been a compelling focus of research for myriad applications. Next‐generation batteries seek to supplant the conventional Li‐ion battery with safer, cheaper, and/or higher‐energy density materials. In the interest of humanoid robotics, increasing volumetric energy density and specific energy of cells via higher energy capacity and operating voltage of electrochemically active materials are of paramount importance. Though lower abundance and high demand yields higher prices for Li than Na, as the lightest alkali metal with a winning standard electrode potential ($E^{\circ }=$ –3.04 V), Li metal and most Li compounds and alloys offer superior power and energy densities. McNulty et al. [66] expertly reviewed current and imminent battery materials for general autonomous mobile robots (AMRs), affirming ‘'there is no ‘one size fits all’ battery for AMRs.' For humanoid robots in particular, however, the following discusses candidate next‐generation battery materials that emphasize specific energy, balanced with stability, and potential cell form factors, highlighting the challenges in their realization.

#### 4.2.1 Cathodes

Further improvements to current generation Li‐ion battery cathodes delve beyond Ni‐rich NCM/NCA (80% Ni) into ultra‐high‐Ni layered lithium transition metal oxides (90+%), whereas next‐generation cathodes are derived from high‐capacity lithium–manganese‐rich (LMR) oxides, alternative olivine/polyanion materials, and high‐voltage spinel LiNi_0.5_Mn_1.5_O_4_ (LNMO). These are all contingent on overcoming issues such as oxygen redox at higher potential and electrolyte stability windows.

Alternative olivine structured LiMPO_4_—where M = Ni, Co, or Mn—has a higher voltage than LFP, but strong distortion of Mn and Ni octahedral bonding coordination in the crystalline lattice induces repeated volume changes every charge/discharge, degrading the material. Established lithium manganese iron phosphate (LMFP) cathodes successfully substitute a portion of Fe with Mn, leveraging Mn redox at a higher voltage to bridge LFP toward NCM energy density, at ∼240 Wh kg^−1^. The distortion of Mn octahedra bonding introduces the compromises in stability and limits the reasonable composition to ∼30% Mn. The spinel structure LiM_2_O_4_ for M = Mn or Ni suffers similar destabilizing effects. And yet LiNi_0.5_Mn_1.5_O_4_ (LNMO) provides a prospective high‐voltage (ca. 4.7 V vs. Li/${\mathrm{Li}}^{+}$) cathode material and theoretical capacity of 147 Ah ${\mathrm{kg}}^{-1}$. These higher voltage batteries are inhibited by electrolyte oxidative stability at the cathode interface. Appropriate engineering of the cathode surfaces and electrolytes may then enable this high‐voltage CAM, still only managing ≈691 Wh kg\rlap^−1^_CAM_.

High‐capacity cathode materials include lithium‐manganese‐rich (LMR, e.g., Li_2_MnO_3_) type materials, with an excess of lithium. With 33% greater Li content compared to LiMO_2_, LMR cathodes effectively artificially inflate theoretical capacity by 33%. The charge balance pushes Mn to nominally ${\mathrm{Mn}}^{4+}$ and above, ideally suppressing issues of ${\mathrm{Mn}}^{3+}$. Unfortunately, lattice oxygen redox—which lends a possibility of high operating voltages >4.2 V—tends to lead to irreversible destabilization of the material. As such, significant steps in ensuring reversible redox mechanisms without oxygen evolution are essential to improve cycle life of this material type. If the larger Li content of LMR‐based materials become usable without rapid degradation, this class of cathode would exceed 1000 Wh kg\rlap^−1^_CAM_.

#### 4.2.2 Liquid Electrolytes

As cathodes are developed for high‐voltage regimes, they must be paired with appropriate electrolytes. Charging too high or too low voltage can push any electrolyte beyond its electrochemical stability window. Various alternatives to conventional electrolytes with different solvents or additives can help lessen degradation and side‐reaction at the anodic or cathodic interfaces, which otherwise irreversibly and hazardously harms the battery at voltage extrema.

#### 4.2.3 Anodes

Much like Si and SiO_x_, several metals and compounds can incorporate Li into their structure. Other alloying anodes, particularly lithium‐indium, are of great interest for next‐generation and all‐solid‐state batteries. Lithium alloy anodes offer high specific energies, but their greatest challenges to these anodes remain: volumetric expansion, SEI/interface stability, and dendrite formation.

Li‐metal anodes provide an ultimate anode density (specific capacity 3860 Ah kg\rlap^−1^_anode_) for lithium‐ion batteries by removing extraneous elements and components of the anode, such as conductive additive and binder. The challenge of Li‐metal anodes is that the lithium can cause shorts as dendrites grow during charge or loss of capacity via “dead lithium” as branches or whole dendritic trees disconnection during discharge [67, 68]. Realization of the lithium metal anode requires consistent, uniform deposition of the lithium‐metal during charge, and uniform planar stripping of lithium during discharge.

#### 4.2.4 All‐Solid‐State Batteries

Improved capacity and safety are two attractive attributes of the all‐solid‐state battery. Replacing the polymer separator and organic liquid electrolyte with a solid‐state electrolyte seeks to maximize active components in the cell, reducing weight and volume. Various approaches include (in)organic polymers, inorganic ceramic compounds, or glassy electrolytes.

Inorganic solid electrolytes—conventionally ion conducting ceramics—encompass numerous potential compounds [69]. Superionic conductors are a baseline threshold for candidates, approaching the ionic conductivity of conventional liquid electrolytes, ca. 1–20 mS ${\mathrm{cm}}^{-1}$. Crystalline solid electrolytes present potential challenges due to grain boundaries, which may raise leakage current and self‐discharge, as well as nucleation and growth of metallic Li, spawning dendritic growths that can short the battery. Glassy electrolytes, with an amorphous nature, will have no grain boundaries, and so will lessen these issues. As either crystalline or glassy inorganic electrolytes are generally ceramic, they are often stiff and brittle. This can introduce issues in fabrication and maintaining contact with the electrodes during operation, given volumetric changes that can accompany the magnitude of mass transport between anode and cathode. Several MPa of applied stack pressure is the common solution to maintain contact and suppress dendrite growth, limiting practicality. Much research effort is directed toward reducing this requirement [70].

The distinction between polymer electrolytes and gel electrolytes is fuzzy, where the latter traditionally includes LiPo (lithium polymer) batteries. Simply put, these sorts of electrolytes include salt additives of the ion of interest (${\mathrm{Li}}^{+}$, ${\mathrm{Na}}^{+}$, etc.) into a polymeric base, can enable ion conductivity through the polymer, particularly enhanced with ionic side chains [23]. Polymer electrolytes can complement inorganic solid electrolytes in a bi‐layer or tri‐layer composite form, where the softer polymer electrolyte lies between an electrode and the inorganic solid electrolyte and conform to each [24]. In addition to mechanical interface benefits, the composite “ceramic in polymer” or “polymer‐in‐ceramic” composite and bi‐layer format can address the challenge of electrochemical stability window [71, 72]. By pairing two distinct solid electrolytes, the composite bridges a wider range of cathodic and anodic stability regions. Pairing a cathode‐facing solid electrolyte (e.g., inorganic ceramic) that has high‐voltage regime stability with an anode‐facing solid electrolyte (e.g., polymer) with low‐voltage regime stability can enable both operating voltage windows and Li‐conductivity properties that are otherwise mutually exclusive.

Li‐alloy anodes have been explored as solutions to the challenges in lithium plating/stripping and contact loss between anode and solid electrolytes. Various solids can incorporate lithium into their crystal structure, much like silicon, leading to alloys with incremental Li contents. Few can do so with much less volumetric change, however. Indium is currently considered by many as the best choice, with high ionic conductivity enabling fast‐charging capabilities. Lithium dendrite formation is still a challenge even here. Attempts to suppress the dendrite growth include engineering of the interface between the anode and solid electrolyte or applying higher external pressure.

#### 4.2.5 Conversion Chemistry Batteries

The pursuit of ultra‐high energy density requires an additional overhaul of battery chemistry and with it, possibly geometry. In contrast to intercalation chemistry of the conventional layered oxides or graphite, conversion chemistry batteries use more energetic reactions. The most mature examples include nanostructured transition metal oxides like Co_3_O_4_, Fe_2_O_3_, or NiO [73]—which are rivaled by Li‐air, Al‐air, Zn‐air, and Li‐sulfur (Li‐S) batteries [27, 28, 74].

Li‐sulfur batteries use a lithium metal anode and bulk sulfur cathode. During discharge, lithium ions react with the sulfur S8 polymeric chains, breaking the chains into smaller Li‐S_x_‐Li chains, for x from 8 to 1. As so much lithium is involved in the complete reaction (Li_16_ + S_8_ → 8Li_2_S), the energy density is on the order of 2600 Wh kg\rlap^−1^_CAM_, and full cell energy density currently managing around 500 Wh ${\mathrm{kg}}^{-1}$ [27]. As covalent/ionic bonds, rather than intercalation into a van der Waals gap, much more energy is involved in each bond, presenting more complex reaction kinetics requiring catalytic activations. Most detrimental to the realization of Li‐S batteries, sulfur compounds that incidentally migrate from the cathode to the anode can react with the lithium metal, poisoning the surface—a so‐called “shuttling” effect. Furthermore, as shown in Figure 3B, the volumetric energy density of Li‐S cells falls short of lithium‐air and solid‐state batteries.

Li‐air batteries use the oxygen from air to oxidize ${\mathrm{Li}}^{+}$ ions at the cathode to form Li_2_O_2_ [75, 76, 77]. The change in form factor and lack of significant mass at the cathode—often high surface area carbon, upon which Li_2_O_2_ grows—enable high energy densities above 3460 Wh ${\mathrm{kg}}^{-1}$. Reversibility of this mechanism is the greatest challenge to overcome.

In many aspects, hydrogen fuel cells are comparable to Li‐air batteries. The lightest ion, $H^{+}$ would be a prime candidate for high energy density, if not for the challenge in storing compressed H_2_ gas or managing highly reversible metal‐hydride storage for fuel. As an alternative to choosing the lighter ions, other non‐alkali metals present chemistry facilitated by more charge transfer per ion. Fe‐air and Al‐air batteries (AAB) involve 3$e^{-}$ reactions and Zn‐air 2$e^{-}$ per ion [78, 79]. Contrary to Li‐air batteries, in which Li^+^ ions carry charge from the anode to cathode, Al‐air and Zn‐air may be mediated by OH^−^ in an alkaline solution. Self‐corrosion at the Al surface to Al_2_O_3_ with accompanying hydrogen evolution reactions (HER) must be addressed to realize AAB technology. Tradeoffs of these include more abundant cations for lower cost than Li‐air, while Fe and Zn are safer and more reversible but possess low specific energy. High theoretical specific energies of Li‐air and Al‐air batteries are hindered by kinetics and side reactions present challenges to yet to be addressed for immature technologies but they may at some point present cheaper alternatives to Li‐ion dominance. In a similar line of thinking, for applications where breathing batteries are not viable, Al‐S batteries may yet be investigated for a viable analog to Li‐S batteries [80].

#### 4.2.6 Primary Battery Chemistry

High energy density Li primary batteries are available for biomedical and defense applications, including theoretical cathode capacities of Li/CF_x_ with > 2000 Wh ${\mathrm{kg}}^{-1}$, Li/SOCl_2_ with 1470 Wh ${\mathrm{kg}}^{-1}$, and Li/MnO_2_ with 1005 Wh ${\mathrm{kg}}^{-1}$ [81, 82, 83]. Specifically, the ideal cathode reaction of Li/CFx batteries provides 865 mAh $g^{-1}$ capacity with 4.5 V of LiF formation reactions. Current practical battery operation is limited by intermediate CLi_x_F graphite intercalation compound formation with lower potential of 2.5–2.8 V [84]. It may be possible to utilize a larger portion of the ideal theoretical energy (865 Ah ${\mathrm{kg}}^{-1}$ × 4.5 V = 3893 Wh ${\mathrm{kg}}^{-1}$) of Li/CF_x_ batteries through a systematic engineering of cathode reactions. Current and future optimized primary batteries would provide a complementary energy source for humanoid robot operation under diverse environments.

### 4.3 Electrode Design

Reiterating the performance and safety needs of humanoid robot batteries, co‐optimization for an energy–power–stability balance will rely on electrode engineering, electrolyte additives for high‐rate stability, and in situ diagnostics tailored to dynamic loads common in robotic systems. In traditional cell design, maximizing energy density requires optimized electrochemically active materials (cathode active materials e.g., NCM, LNMO, LFP; anode materials e.g., graphite, silicon, Li‐metal) and minimizing inactive components (polymer binders, conductive additives, and current collector, and polymer separator).

Thick coated electrodes with low percentages of conductive additives and binders enable maximum CAM and active anode mass loading. Thicker electrodes (translating to mass loading, [g_active_ ${\mathrm{cm}}^{-2}]$) can fill the battery pouch or cylinder can with fewer layers or wrappings, thus reducing layers of current collector and separator, which consequently reduces volume for liquid electrolyte to fill. Kim et al. [21] outlined the challenges of very thick electrodes for high‐areal‐capacity battery electrodes, including up‐scaled manufacturing (roll‐to‐roll or R2R coating) difficulties and charge transfer of ions and electrons within the electrodes. Thinner separators can inversely leave more room for electrode in the same cell volume, to improve volumetric energy density and reduce internal resistance to enable higher power density.

Composite electrodes such as graphite‐Si or graphite‐SiO_x_ anodes should optimize coating and calendering to densify. Remaining porosity of the casted electrode can provide room for volumetric expansion of such components. Alloy electrodes may be fabricated by distinct R2R methods from thinning of ingots or by chemical vapor deposition (CVD) onto current collectors. Grain orientations and porosity to control/alleviate stresses from volume expansions present significant challenges to commercial manufacturing at the electrode design level [65, 85].

Dry electrode technology could lower manufacturing cost by reducing the energy requirements for drying solvents from a wet slurry, but dry electrode manufacturing is far from optimized [21, 86]. A bifunctional component, like a conductive binder could similarly impact the relative active material (c) by a couple percent. Conductive polymers of LiPo batteries and solid‐electrolytes are nominal bifunctional separators. Lee, et al. [21] suggests electrode porosity below 30% and active material content above 96 wt.% for areal capacity above 5 mAh ${\mathrm{cm}}^{-2}$. An anode (negative, N) to cathode (positive, P) capacity N/P ratio below 1.2 and electrolyte fill < 2.5 g ${\mathrm{Ah}}^{-1}$ can help balance energy/power density for cell performance and safety.

### 4.4 Battery Design

Beyond the cell electrodes, the electrolyte, separator, and cell container components optimize battery internals' usage. Then, the form factors of battery cells contribute directly to pack design and packing efficiency $p(n_{c})$. Larger diameter cylindrical cells have been pursued, moving from 18 650 and 21 700 to 4680 (46 800) and 4690 (46 900) to maximize capacity per cell and minimize inactive shell material. Packing of cylinders is independent of diameter, so decreasing the ratio of cylindrical cell shell thickness cell diameter and designing pack containers based on packing types (hexagonal packing of circles optimally approaches 90.7%) as a bounded area (e.g., 20 circles in a square: ≤ 80%) dictate bulk optimization of packing cylinder cells.

The battery technologies essential to reaching 10 kWh pack energy in 10 L and/or 20 kg require >1000 Wh L^−1^/500 Wh kg^−1^: solid‐state electrolytes, Li‐metal or Li‐alloy anodes, and/or conversion cathodes. These each require unique adaptations to established Li‐ion fabrication methods. Current prospects of next‐generation all‐solid‐state batteries are most presently suited to packs of pouch cells, where applied stack pressures ensure good contact between electrodes and electrolytes [87]. Cylindrical cells with spring core mandrels could regulate internal pressures, but cylinder cell fabrication methods would further emphasize the requirements for solid electrolytes to be thin and flexible. Mechanically softer electrolytes may be such a solution, and may also lower required stack pressure, enabling thinner cylinder cell cans or utilization of aluminum for better heat transfer without critically compromising mechanical strength. Meanwhile cylindrical or prismatic form factors may be sufficiently suitable for Li‐S batteries, pouch cells would be favored for the lowest packaging weight [88]. Various metal‐air batteries require prismatic‐like cells to engineering air cathode surface area, while notable extra infrastructural implementations at the system level must ensure proper O_2_ levels in the ambient air supply, and further integration challenges to leverage a refreshable anode [8].

### 4.5 System Design

To meet many operating requirements for humanoid robot workloads, swappable battery packs and/or next‐generation batteries will still be required. Lessons from the EV industry point to blade batteries by BYD, incorporating prismatic cells directly into the pack frame to minimize additional space and structural needs. A hot‐swappable pack module that integrates with the humanoid robot BMS and TMS infrastructure (significant challenges) is the most promising solution for continued robot functionality during battery recharge, as well as enabling high‐capacity non‐rechargeable batteries or conversion battery chemistries with intensive recharge procedures.

The mission or task parameters and environment equally dictate broader humanoid robot design and the battery within.

In industrial, warehouse, or medical care scenarios, low‐capacity and swappable packs are most suitable with ready access to charging. Excess packs negate charging downtime at much more accessible costs than fleets of battery‐embedded robots.

More dynamic in‐the‐field scenarios, such as emergency/rescue, may prioritize high specific energy for thermal management overhead (extreme weather demands), long operation times, and less predictability. In these scenarios, swappability is crucial as well, as charging infrastructure may be unavailable. Primary batteries may suit these applications for maximum operation times in emergency scenarios.

As next‐generation technologies develop, distributed embedded batteries will enable highly robust energy solutions for diverse use cases.

### 4.6 Artifical Intelligence and Humanoid Robot Batteries

Artificial Intelligence (AI) and humanoid robots are inextricably linked in myriad facets. Humanoid robots may inevitably serve as a physical host body for AI, allowing physical interaction and integration with the human world [52]. Conversely, AI algorithms in present and near‐term forms may play essential roles in realizing the above technological developments.

Throughput and reproducibility are significant hurdles in materials discovery and validation. Machine learning and AI‐driven labs and computational systems for material exploration and development are in their earliest development now [89, 90]. Large parameter spaces in material synthesis routes and environmental variables obfuscate battery material performance. Bayesian optimization techniques to guide exploration of parameter spaces must develop toward automation of workflows and incorporation of physics‐informed closed‐loop AI platforms to more robustly optimize battery materials. Furthermore, atypical experimental designs—expanding beyond human‐friendly conditions—could unlock previously under‐explored regimes of chemistry. Advanced manufacturing techniques to scale next‐generation battery materials commercially include in‐line characterization such as metrology during fabrication of cells and packs. Development of systems to optimize pilot‐scale processing conditions from live data or incorporate into digital twins will similarly benefit from physics‐informed ML/AI systems.

Worker health and infrastructure costs surrounding safe material handling are additional hurdles to commercialization. While current generation NCM cathodes require rather dry conditions (<1% RH, dew point –40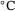 at 60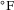) when CO_2_ is present to avoid degradation, liquid electrolytes require the same to mitigate reaction between the fluoride in dissolved lithium salts and water, which can produce hydrogen fluoride (HF) gas [91]. Similarly, sulfur and many solid electrolytes including sulfide and halide compounds can undergo hydrolysis, releasing corrosive H_2_S, HF, or HCl vapor and degrade the materials [92, 93]. Lithium metal reactions with water yield flammable‐hazard H_2_ gas. It seems inevitable that next‐generation battery fabrication will require ultra‐dry environments, dew point below –60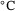 [92, 93]. As human bodies are often the limiting factors in maintaining an operating dry room's dew points, development of these battery materials through pilot‐scale cell fabrication may come to rely on embodied AI.

AI monitoring of robotic system sensors for mobility, task planning, TMS, and BMS that require sophisticated logics will enable optimized performance of humanoid robots and their energy systems [1]. Ultimately, AI today helps to optimize these technologies for AI beneficiaries of tomorrow.

## 5 Summary

The challenges presented in realizing humanoid robots are intimately interlinked. Integrating power solutions within an intricate network of electronic and mechanical components, while managing heat (via TMS), state of charge/state of health (SoC and SoH via BMS), and more, is a multi‐dimensional and interdisciplinary challenge. As battery technologies evolve, greater performance may be enabled with more forgiving operating parameters or redesign to accommodate unconventional chemistry and form factors.

As the demands for higher energy density batteries escalate, humanoid robots will require greater capacity and/or higher operating voltage, to achieve energy needs of 10+ kWh. Theoretical limits to high‐nickel NCM cathodes with liquid electrolytes or LiPo have finite room to improve. As innovations develop to mitigate degradation and unlock higher capacity/voltage of Ni‐rich or LMR type cathodes, Li‐ion batteries will continue to incrementally increase energy densities over the next few years. To enable a leap to higher voltages in the conventional formats, development of liquid electrolytes for wider electrochemical stability will be necessary. On the anode side, supplementing graphite with silicon for silicon–graphite composites will continue increasing energy density as well.

True leaps in gravimetric energy density will follow realization of next‐generation batteries, from enabling (a) lithium‐metal anodes, and (b) all‐solid‐state batteries. Lithium metal batteries will maximize the operating voltage while minimizing anode mass and inactive components, with moderate volume expansion. Implementing thin solid electrolytes tends to decrease mass and volume, while also helping to enable lithium‐metal or ‐alloy anodes—once material and fabrication challenges are overcome. These technologies may contribute to enabling conversion chemistries for further revolutionary increases in volumetric and/or gravimetric capacities on the order of 1000–10 000 Wh $L^{-1}$ or Wh ${\mathrm{kg}}^{-1}$. Robotics in field scenarios without options to recharge may rely on advanced primary batteries, requiring swappable battery schemes.

The unique challenges of humanoid robot batteries fall to optimizing centralized, swappable batteries or distributed, embedded batteries. Efficient integration of thermal management systems and sophisticated battery management systems for maximizing system life and mechatronic performance must consider the battery pack, cell, and material health. A fourfold increase in energy density to 10 kWh would enable a half‐marathon on current mechatronic systems. Matching this with similar advances to mechanical and compute system efficiency, a full eight‐hour shift of manufacturing should be achievable. These improvements are critical to realize cohesive integration of humanoid robots in humanity's industry and world.

## Conflicts of Interest

The authors declare no conflicts of interest.

## Data Availability

The data that support the findings of this study are available from the corresponding author upon reasonable request.
